# Challenges and perspective of cancer patients during the COVID-19 pandemic and the Lebanese financial crisis: a multicentric cross-sectional study

**DOI:** 10.3332/ecancer.2022.1359

**Published:** 2022-02-24

**Authors:** Rashad Nawfal, Jad Kassem, Hassan Salame, Ali Kassem, Pascale Salameh, Bassam F Matar

**Affiliations:** 1 Faculty of Medical Sciences, Lebanese University, 3 Campus Lebanese University - Hadath, Beirut, Lebanon; 2 Department of Internal Medicine, Faculty of Medical Sciences, Lebanese University, 3 Campus Lebanese University - Hadath, Beirut, Lebanon; 3 Department of Surgery, Faculty of Medical Sciences, Lebanese University, 3 Campus Lebanese University - Hadath, Beirut, Lebanon; 4 INSPECT-LB: Institut National de Santé Publique, Épidémiologie Clinique et Toxicologie-Liban, Beirut 00000, Lebanon; 5 Department of Primary Care and Population Health, University of Nicosia Medical School, Nicosia 2408, Cyprus; 6 Faculty of Pharmacy, Lebanese University, 3 Campus Lebanese University - Hadath, Beirut, Lebanon; 7 Department of Internal Medicine, Faculty of Medical Sciences, Division of Haematology and Oncology, Lebanese University, 3 Campus Lebanese University - Hadath, Beirut, Lebanon; a https://orcid.org/0000-0001-5428-5660; b https://orcid.org/0000-0002-3804-6173; c https://orcid.org/0000-0002-4780-0772

**Keywords:** COVID-19, cancer, knowledge, challenges, delivery of health care, Lebanon

## Abstract

Lebanese cancer patients are facing a war on two fronts, between the COVID-19 pandemic and one of the most severe financial crises globally in recent times. This multicentric cross-sectional study was conducted, aiming to analyse challenges and perspective of this particularly vulnerable population. A questionnaire was prepared to assess socio-demographic data, challenges faced during the pandemic, perspectives concerning cancer and COVID-19, a scale was also validated to assess knowledge level regarding COVID-19 in this population. Patients were interviewed in chemotherapy units from four different private and public hospitals in Lebanon during December 2020 and March–June 2021. In total, 272 patients were interviewed (median age, 57 years, range, 22–90 years). Concerning salary during the pandemic, it changed negatively (45.6%), or did not change (7.7%), while 46.7% of participants did not work. Some challenges such as transportation problems (39%), inability to reach their physician by phone (16.9%), deferral of chemotherapy dates (31.6%), difficulty finding chemotherapy medications (49.6%) were more likely to be reported by patients receiving chemotherapy in public compared to private hospitals (*p* < 0.01 for each). Other challenges include not being able to find non-cancer-related medications (71.3%), this challenge being increased when comparing December 2020 with March–June 2021 (*p* < 0.02). Using a multivariate analysis, the best predictor for increased knowledge about COVID-19 was higher levels of education (*p* < 0.001). In conclusion, this study shows that cancer patients in Lebanon are facing many challenges that complicate different aspects of health. Perspective and challenges of these patients must be taken into consideration in order to deliver better care to our patients in these unprecedented times.

## Background

The severe acute respiratory syndrome coronavirus 2 (SARS-CoV-2) virus has resulted in an ongoing pandemic. At the time of writing (August 2021), and according to the World Health Organization, over 215 million people have developed the resulting illness, COVID-19, causing more than 4.48 million deaths worldwide. Most people have been either directly or indirectly affected by this pandemic in many ways from healthcare to the economy. However, there are certain subgroups such as cancer patients that have been disproportionately affected, as according to multiple studies [1, 2], cancer patients tend to have worse outcomes when infected with SARS-CoV-2.

Moreover, cancer patients are at a high risk of immunosuppression that can be caused by the malignancy itself or the treatment. Lack of immunity makes these patients prone to serious infections, especially COVID-19, that can aggravate their condition and increase the rate of unnecessary hospitalisation that can delay the treatment of cancer, hence affecting the prognosis negatively [3]. Patients that suffer from haematologic cancer and those who are receiving chemotherapy are at a higher risk of immune deficiency [4]. In comparison to the general population, the rate of contracting SARS-CoV-2 in cancer patients is higher according to some reports, especially in male gender and haematological malignancies [5, 6], and frequently leads to a worsened condition and a very poor prognosis [7]. A study analysed health records of the US Veterans Affairs Healthcare System for the prevalence of COVID-19 infection in cancer patients. Of 22,914 cancer patients, a higher prevalence of COVID-19 infection was found in people with haematologic malignancies compared to solid tumours (10.9% versus 7.8%,* p* < 0.001) [5].

Cancer by itself is one of the main causes of death globally, and its burden is considerable in countries of all income levels. However, this burden can be decreased through proper treatment and comfort care provided by the country and internationally [8]. Healthcare related to cancer may however be reduced due to the COVID-19 situation, since hospitals are getting more and more overwhelmed and saturated from the current situation. The COVID-19 pandemic is not only delaying the treatment of malignancies, but also new diagnosis. Some haematologic malignancies and solid tumours need rapid intervention for treatment or diagnosis, other malignancies do not [9].

In Lebanon, there has been a total of 8,031 deaths thus far (26th of August 2021, Ministry of Public Health), with a reported death rate of 1,172/million person, which has led to a shortage in hospital beds and medical resources [10]. In addition, Lebanon is currently experiencing the third most severe financial crisis worldwide since the mid-19th century according to the World Bank [11], with a money depreciation of more than 90%. There is a massive shortage of medications which led to many cancer patients having a hard time accessing chemotherapy as well as other drugs, the wealthiest of patients are buying their medications from abroad [12]. Cancer patients in Lebanon are facing a ‘war on two fronts’. The first front is facing one of the most severe financial crises and medication shortages in recent times, and the second is facing the COVID-19 pandemic and the potential implications of this pandemic on the evolution of their cancer.

The aim of this study was to uncover the challenges faced by cancer patients in Lebanon during this critical period, assess level of knowledge regarding COVID-19 and find predictors of increased knowledge, as well as to assess different perspectives concerning the novel coronavirus and their malignancy.

## Methods

### Study design

This is a cross-sectional, multicentric study, conducted in multiple private and public chemotherapy units, from rural and urban areas in Lebanon. Namely, Rafik Hariri University Hospital, Al Zahraa Hospital University Medical Center, Dar al Amal University Hospital and Saint Charles Hospital.

In total, 272 cancer patients aged between 22 and 90 consented to participate in this study. Thirty patients were interviewed at Dar al Amal University Hospital during December 2020. The rest were interviewed between March 2021 and June 2021 in all four chemotherapy units included in this study. Patients were interviewed by the investigator who was recording all responses on the questionnaire form. The interview was performed in Arabic, and then the questionnaire was translated by two professional translators. After making sure that both versions matched perfectly, answers were filled on the translated English version for all participants.

The questionnaire consisted of four parts: the first part asks about socio-demographic data. The second part asks about challenges faced by cancer patients. The third part is a knowledge scale we validated to use on this specific population. Finally, the fourth part asks about perspectives regarding COVID-19 and their malignancy.

### Validation and reliability of the scale

The part aiming to assess knowledge level regarding COVID-19 is a 3-point Likert scale ranging from 0 = disagree, 1 = don’t know, 2 = agree. ‘Don’t know’ was added as an answer in order to offer a middle response alternative to people who do not possess a clear-cut opinion on the statement provided [13]. Statements (2, 4, 5, 6, 7 and 8) were scored in reverse order of the above-mentioned scoring as the correct answer for these statements was ‘disagree’.

Initially, the literature review was conducted on the Centers for Disease Control and Prevention and the World Health Organization’s websites to develop a data pool of some common statements and misconceptions regarding COVID-19, which was then reviewed and approved by two oncologists and an expert in questionnaire formulation. After reviewing and corrections, we narrowed down the questionnaire to eight final statements aiming to assess knowledge level regarding COVID-19 (See Supplementary File).

Validation of the questionnaire was assessed by ways of discriminant validity, face validity and construct validity. Discriminant validity was assessed using Fornell–Larcker criterion suggested in 1981 for assessing discriminant validity [14]. According to this criterion, if the square root of the Average Variance Extracted of our two components is greater than the correlation coefficient between component 1 and component 2, then the model satisfies the discriminant validity criterion. Pearson’s correlation between our two components yielded a value of 0.297 with *p* < 0.001. The square root of the Average Variance Extracted of our two components was equal to 0.698. Meaning that our scale presents an adequate discriminant validity.

Face validity was established by the experts as well as the participants who stated they had no difficulty understanding and answering to all eight statements. To assess Construct validity, a Principal Component Analysis was performed, and yielded a Kaiser–Meyer–Olkin value of 0.785 which is satisfactory, and the Bartlett’s test for sphericity was significant (*p* < 0.001) indicating that the factor analysis is suitable.

**Figure 1.**
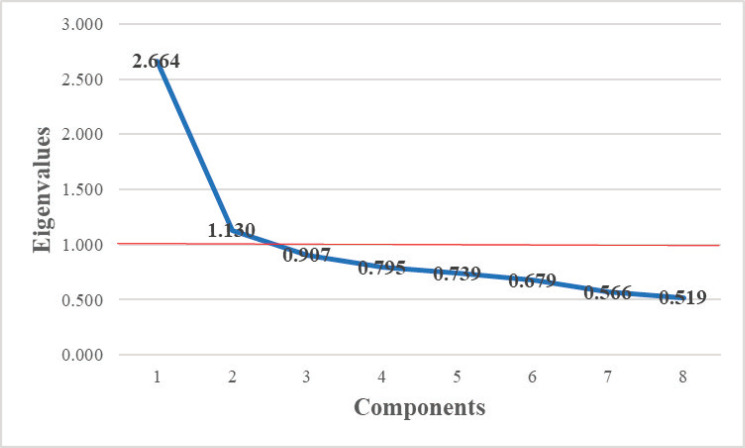
Scree plot for factor identification.

Two components were then extracted for Principal Component Analysis, both above the eigenvalue limit set at 1.0 according to the scree plot for factor identification (see Figure 1). The percentage of total variance explained by these two components was 47.427%, component 1 explains 33.303% and component 2 explains 14.124% of the total variance. Factor 1 represents erroneous statements, and factor 2 represents true statements. Rotation was done using Varimax with Kaiser normalisation, all items had factor loadings on a single factor greater than 0.3 and ranging from 0.403 to 0.823 (see Table 1) which is acceptable.

As to sample size, the acceptable ratio of the number of people to the number of measured variables should exceed 10 to perform exploratory factor analysis. Our questionnaire was completed by 272 cancer patients in total, providing a ratio of 34 in total to our scale containing 8 items, meaning that our sample size is adequate.

Reliability was established using Cronbach’s alpha on the final scale answers, a score of 0.7 and higher can be considered as acceptable [15]. Cronbach’s alpha for this scale was equal to 0.711.

### Inclusion and exclusion criteria

Inclusion criteria included cancer patients from multiple oncology clinics receiving chemotherapy treatment in any of the four chemotherapy units and during the timeframe this study was conducted in. All included participants completed the consent form after having it thoroughly explained by an investigator.

The exclusion criteria included non-cooperative patients, patients who do not reside in Lebanon, and minors under 18 years of age.

### Statistical analysis

Statistical analyses were carried out on R software version 4.0.5 (R Foundation for Statistical computing). Descriptive statistics were reported as median, interquartile range and range for age; median, interquartile range, range and Standard Deviation for knowledge score, and frequency with percentages for categorical variables. Categorical variables were described using counts and percentages. Age and total score of knowledge towards COVID-19 were categorised, each, into two groups with their medians (57 and 9, respectively) serving as the cut-off values. Univariate logistic regression was used to assess the association between the presence of challenges (dependent variable) and the funding type of the hospital where patients have received chemotherapy (public versus private as independent variable). Multivariate logistic regression analysis was conducted to assess the factors that are associated with the knowledge score being above or below average. Only factors with a *p*-value below 0.2 on Chi-squared test were included in multivariate logistic regression. The model was not found to be badly fitted on Hosmer–Lemeshow goodness of fit test for multivariate logistic regression (*p*-value = 0.132). For logistic regression analyses, odds-ratios, 95% confidence interval for odds-ratios and *p*-values were reported. Statistical significance level was set at *p*-value < 0.05.

### Ethical considerations

The research proposal was first approved by the ethics committees of all medical centres involved in this study. Institutional Review Board (IRB) approval was granted from Rafik Hariri University Hospital on the 1st of March 2021 as well as from Al Zahraa Hospital University Medical Center with reference number 4/2021. A written informed consent form was also completed by all the participants. An Investigator was present at all times, and explained the anonymity and confidentiality of the data as well as patients having the right to drop out of the study at any time without any retributions or repercussions.

## Results

### Socio-demographic data

Of the 272 patients who participated in this study, 37.1% were males and 62.9% were females, median age was 57 years, interquartile range was 46–67 and range was 22–90. The percentage of patients who were infected and recovered from COVID-19 before participating in this study was 25%. Concerning the funding type of the hospital where patients received chemotherapy, 19.9% of patients received their treatment in a public hospital (Rafik Hariri University Hospital), the remaining 80.1% of patients at Private hospitals. 42.6% of participants received their treatment outside the Greater Beirut area in Baalbek (Dar Al Amal University Hospital) and the remaining 57.4% inside the Greater Beirut area. Regarding the intent of treatment, it was curative in 94.8% and palliative in 5.2%. The summary of socio-demographic data is shown in Table 2.

### Challenges faced during the pandemic

The most commonly faced challenge by participants in this study was not being able to find non-chemotherapy drugs such as Hypertension and Diabetes medications, Antibiotics or Non-steroidal anti-inflammatory drugs (71.3%). Prevalence of various challenges assessed in this study can be found in Table 3.

After comparing the prevalence of all challenges based on the funding source of the hospital, it can be noted that ‘transportation problems from residence to the hospital’ (39% of all participants), ‘not always being able to reach their physician online by phone calls or WhatsApp’ (15.8% of all participants), ‘deferral of radiotherapy/chemotherapy dates’ (31.6% of all participants) and ‘not always being able to find chemotherapy medications’ (49.6% of all participants) were all challenges more likely to be observed in Public compared to Private hospitals (*p* < 0.001, *p* < 0.001, *p* < 0.01 and *p* < 0.001, respectively). The full comparison between the prevalence of challenges in Private and Public hospitals can be found in Table 4.

### Knowledge of cancer patients regarding COVID-19

In regard to responses to the scale to assess knowledge level towards COVID-19, median score was 9, standard deviation was 3.68, interquartile range was 6–12 and range was 1–16. The most common misconception in this population was that garlic could protect from contracting COVID-19 (48.5% answered Wrong), other common misconceptions include that ‘hot beverages could protect from contracting COVID-19’ (44.5% answered Wrong), and that ‘COVID-19 can be transmitted through the water of swimming pools’ (40.8% answered Wrong). A description of the items and answers to this scale can be found in Table 5.

Concerning the multivariate analysis to find predictors of increased knowledge level regarding COVID-19, all variables included in the analysis had a *p*-value < 0.2 in bivariate analysis. Variables included were age, intent of treatment, prior diagnosis of COVID-19 infection, education status, funding source of the hospital offering treatment, level of worry towards contracting COVID-19, fear of cancer compared to COVID-19 and awareness that chemotherapy could decrease immunity and increase health-related risks due to COVID-19. The best predictors were higher levels of education, such as being a High School Graduate (*p* < 0.001) and a College Graduate (*p* < 0.001). Results of the multivariate analysis can be found in Table 6.

### Perspective regarding COVID-19 and cancer

Concerning the reported level of worry regarding COVID-19, 30.9% reported a minimal level of worry, 31.6% reported an average level of worry and 37.5% reported a maximal level of worry. As to fear from cancer and COVID-19, 24.3% reported that they feared COVID-19 more than cancer, 61.8% reported that they feared cancer more than COVID-19, 11.4% feared both equally and 2.6% stated that they feared none. As to the knowledge that chemotherapy could lower the immunity and thus increase the chance of health-related risks due to COVID-19, 75.7% reported that it could, 22.1% reported that it could not, and 2.1% did not know. Finally, in regard to preventive measures at home against COVID-19, 13.2% used face masks and social distancing only, 23.9% did self-quarantine at home only, 28% did both of the above, and 34.9% did none of the above. The full results of perspectives regarding COVID-19 and cancer can be found in Table 7.

## Discussion

This study is the first of its kind to assess the challenges, as well as the perspective and knowledge concerning COVID-19 of cancer patients during the Lebanese financial Crisis and concomitant COVID-19 pandemic. In total, 62.9% of participants in this study were women and median age was 57. This finding was expected as breast cancer is one of the most common malignancies in Lebanon. This type of cancer also presents at a median age of 50 in Lebanon, compared to a median age of 63 years in western countries [16], which could account for the female predominance as well as the median age in this study. As of the end of June 2021, approximately 7.97% of the Lebanese population contracted COVID-19 at some point during the pandemic according to the Lebanese Ministry of Public Health [10]. In this study, 25% of participants had contracted and recovered from COVID-19, which could hint towards an inadequacy in testing for this virus in the general population in Lebanon. Concerning the education status in this population, 13.2% of participants were illiterate which is in concordance with the United Nations Educational, Scientific and Cultural Organization statistics in Lebanon [17]. Finally, in regard to the reported change in monthly income, 45.6% reported a decrease, 7.7% reported no change and 0% reported an increase in monthly income. 46.7% reported they did not work. Meaning the majority of cancer patients in Lebanon have experienced a financial setback during this period.

**Figure 2.**
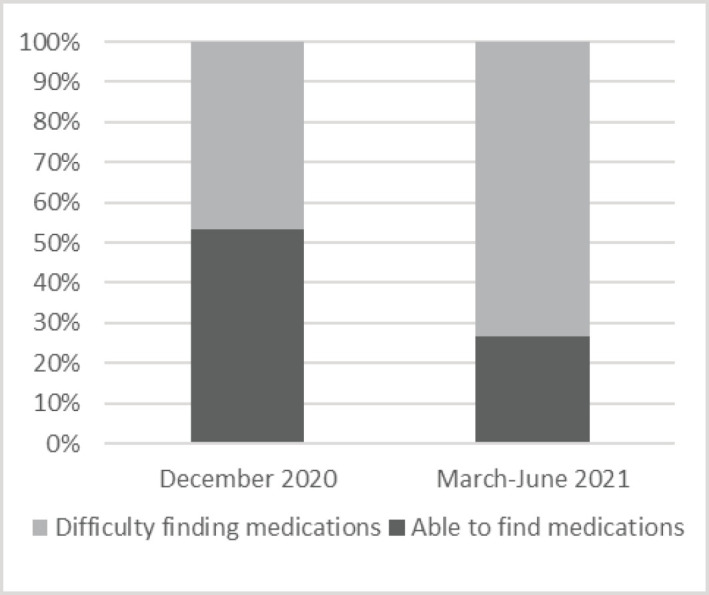
Ability to find other non-chemotherapy medications in December 2020 compared to March–June 2021 (*p* = 0.015).

For challenges faced by cancer patients, a somewhat similar study was done on 36 cancer patients in India by Mitra and Basu [18]. Challenges assessed were transportation from residence to the hospital which was faced by 77.8% compared to 39% in this study. Another similar challenge was non availability of chemotherapy medications which was faced by 22.2% compared to 49.6% in this study. This high prevalence for this challenge was expected as Lebanon is facing a shortage in all kinds of medications, especially chemotherapy drugs [12]. Concerning difficulty finding non-chemotherapy medications such as non-steroidal anti-inflammatory drugs, antibiotics, hypertension and diabetes medications, this challenge was faced by 71.3% of all participants in this study. This challenge also increased during the timeline of the study, when comparing December 2020 with March until June 2021 (*p* < 0.02) as shown in Figure 2.

Concerning the knowledge of cancer patients regarding COVID-19, statements that only a minority of participants had a correct answer for were: COVID-19 can spread through the water of swimming pools (24.6% answered correctly). Eating garlic can protect from contracting COVID-19 (26.9% answered correctly). Hot beverages can protect from contracting COVID-19 (33.1% answered correctly). COVID-19 can spread through food, through the digestive system (37.1%). As well as that COVID-19 is always accompanied by a loss of taste and smell, in 100% of people who contract the virus (40.8%). These misconceptions can be caused by lack of access to information, lack of communication with their physician, they can also be caused by different sources of media spreading misinformation [19], or simply due to inability to recall information.

A study was also done on the perspective of cancer patients on 302 participants from India by Ghosh *et al* [20]. The level of worry regarding contracting COVID-19 was very much in 19% of participants compared to 37.5% in this study, moderate in 45% compared to 31.6% in this study and minimal in 39% compared to 30.9% in this study. Another similar question was if patients feared cancer more that COVID-19 or vice versa, 72% feared cancer more than COVID-19 compared to 61.8% in this study and 26% feared COVID-19 more than cancer compared to 24.3% in this study. Finally, a third similar question was if patients knew that chemotherapy could cause more health-related outcomes due to COVID-19 where 52% answered it could, compared to 75.7% in this study. So, comparing these two populations, we can say that participants in this study reported better knowledge about the immunosuppressive potential of chemotherapy and worse prognosis of COVID-19 infection with *p* < 0.001 on chi-squared test. This could be related to good pre-chemotherapy counselling, better recall of information, or simply due to new research published since conducting this study, which clinicians may have used in counselling, also these relatively new findings being available to be accessed by patients on their own.

Some limitations to this study would include, the tool used to assess knowledge level of cancer patients was adapted and validated specifically for this study instead of using an already used and validated scale. Second, we were not able to perform Confirmatory Factor Analysis (CFA) which confirms whether the extracted factor structure truly and adequately describes the data using different model fit indices. Performing CFA would need a second sample which we were not able to provide due to limitations in time, resources as well as limitations induced by the pandemic. Third, due to the barriers imposed by the pandemic, the sample was not designed to statistically represent the population of all cancer patients in Lebanon and make rigid extrapolations, but to give sufficient insight, for the first time, on the challenges, knowledge towards COVID-19 and perspective of this population during the COVID-19 pandemic and concomitant Lebanese financial crisis. Fourth, due to the Hawthorne effect, patients might have given some answers they might perceive as acceptable since they were interviewed in the hospital. Fifth, this study was made in four specific medical centres, excluding other larger chemotherapy units which might have led to selection bias.

## Conclusions

This study shows that cancer patients in Lebanon are facing many challenges that complicate different aspects of health. The majority of these challenges are found in increased proportions in public compared to private hospitals. Also, some challenges are increasing over the timeline of this study, suggesting that Lebanon is in need of rapid interventions in order to help people in their most vulnerable. Finally, the perspective and challenges of cancer patients must be taken into consideration in order to deliver better care during these unprecedented times.

## Conflicts of interest

The authors declare that they have no conflicts of interest.

## Funding

This research work is not funded by any organisation.

## Authors’ contributions

RN, JK and BFM developed the project idea. RN, JK, PS and BFM developed the questionnaire. RN, JK and AK worked on data collection. RN, JK, HS and PS analyzed the data. RN drafted the paper. All authors critically reviewed the manuscript for important intellectual content and agreed on the final version.

## Figures and Tables

**Supplementary Table 1.** Reliability statistics of the 15 initial items of our pilot study.

**Reliability statistics**	
**Cronbach's Alpha**	***N* of items**
0.683	15

Item-total statistics				
	Scale mean if item deleted	Scale variance if item deleted	Corrected item-total correlation	Cronbach's alpha if item deleted
1- Infected children showing no symptoms can spread the virus to others (Agree)	29.78	20.499	0.334	0.664
2- Runny nose is considered a major symptom of COVID-19 (Disagree)	28.63	19.597	0.343	0.661
3- COVID-19 is always accompanied by a loss of taste and smell, in 100% of people who contract the virus (Disagree)	29.22	19.273	0.291	0.671
4- Diffuse body and throat pain is considered as symptoms of COVID-19 (Agree)	29.88	22.629	0.024	0.700
5- COVID-19 can spread through cough (Agree)	29.97	20.676	0.434	0.658
6- COVID-19 spreads in cold weather and does not spread in warm and humid weather. (Disagree)	28.41	21.733	0.119	0.687
7- Influenza vaccine protects from contracting COVID-19 (Disagree)	28.41	22.055	0.132	0.683
8- COVID-19 can spread through food through the digestive system (Disagree)	29.00	18.903	0.362	0.658
9- Hot beverages protect from contracting COVID-19 (Disagree)	28.94	18.641	0.512	0.636
10- Hair loss is considered a symptom of COVID-19 (Disagree)	28.81	19.770	0.364	0.658
11- Eating garlic protects from contracting COVID-19 (Disagree)	28.69	20.673	0.324	0.665
12- COVID-19 can spread through the water of swimming pools (Disagree)	28.91	19.830	0.349	0.660
13- COVID-19 can spread through mosquito stings (Disagree)	28.53	21.289	0.241	0.674
14- Wearing a face shield without a mask protects from contracting COVID-19 (Disagree)	28.63	19.016	0.427	0.648
15- You should not wear a face mask while exercising at the gym (Agree)	29.53	20.902	0.163	0.687

Agree/Disagree in the parentheses denotes the intended correct answer

**Supplementary Table 2.** Reliability statistics after deleting items 4, 6, 7, 13 and 15.

**Reliability statistics**	
**Cronbach's Alpha**	***N* of items**
0.707	10

Item-total statistics				
	Scale mean if item deleted	Scale variance if item deleted	Corrected item-total correlation	Cronbach's alpha if item deleted
1- Infected children showing no symptoms can spread the virus to others (Agree)	19.06	15.415	0.273	0.699
2- Runny nose is considered a major symptom of COVID-19 (Disagree)	17.91	13.894	0.415	0.676
3- COVID-19 is always accompanied by a loss of taste and smell, in 100% of people who contract the virus (Disagree)	18.50	13.613	0.347	0.692
5- COVID-19 can spread through cough (Agree)	19.25	15.613	0.349	0.692
8- COVID-19 can spread through food through the digestive system (Disagree)	18.28	13.499	0.394	0.681
9- Hot beverages protect from contracting COVID-19 (Disagree)	18.22	13.660	0.485	0.664
10- Hair loss is considered a symptom of COVID-19 (Disagree)	18.09	14.217	0.412	0.677
11- Eating garlic protects from contracting COVID-19 (Disagree)	17.97	15.451	0.286	0.697
12- COVID-19 can spread through the water of swimming pools (Disagree)	18.19	14.738	0.311	0.694
14- Wearing a face shield without a mask protects from contracting COVID-19 (Disagree)	17.91	13.830	0.427	0.674

Agree/Disagree in the parentheses denotes the intended correct answer.

**Table 1.** Rotated component matrix for Principal Component Analysis.

	Component	
	1	2
4- COVID-19 can spread through food, through the digestive system (Disagree)	0.687	
7- COVID-19 can spread through the water of swimming pools (Disagree)	0.679	
5- Hot beverages protect from contracting COVID-19 (Disagree)	0.677	
2- COVID-19 is always accompanied by a loss of taste and smell, in 100% of people who contract the virus (Disagree)	0.675	
6- Eating garlic protects from contracting COVID-19 (Disagree)	0.603	
8- Wearing a face shield without a mask protects from contracting COVID-19 (Disagree)	0.403	
3- COVID-19 can spread through cough (Agree)		0.823
1- Infected children showing no symptoms can spread the virus to others (Agree)		0.713

Extraction Method: Principal Component Analysis

Rotation method: Varimax with Kaiser normalisation

Two components extracted: Factor 1: Erroneous statements; Factor 2: True statements

Agree /Disagree in the parentheses denotes the intended correct answer

**Table 2.** Socio-demographic data.

	*n* (%)
Location of hospitalisation	
Zahraa Hospital University Medical Center	86 (31.6%)
Rafic Hariri University Hospital	54 (19.9%)
Dar al Amal University Hospital	116 (42.6%)
Saint Charles Hospital	16 (5.9%)
Gender	
Male	101 (37.1%)
Female	171 (62.9%)
Intent of treatment	
Curative	258 (94.8%)
Palliative	14 (5.2%)
Time of diagnosis	
Before the pandemic	132 (48.5%)
During the pandemic	140 (51.5%)
Were you diagnosed with COVID-19?	
Yes	68 (25%)
No	204 (75%)
Education status	
Illiterate	36 (13.2%)
Elementary school	132 (48.6%)
High school graduate	68 (25%)
College graduate	36 (13.2%)
Did your monthly income change during the pandemic	
Yes, negatively	124 (45.6%)
Yes, positively	0
No	21 (7.7%)
I don’t work	127 (46.7%)
Site of malignancy	
Solid tumours	223 (81.99%)
Breast	103 (37.9%)
Lung	34 (12.5%)
Colon	24 (8.8%)
Ovary	12 (4.4%)
Stomach	8 (2.9%)
Bladder	7 (2.6%)
Prostate	6 (2.2%)
Kidney	4 (1.5%)
Larynx	4 (1.5%)
Liver	4 (1.5%)
Cervix	3 (1.1%)
Cholangiocarcinoma	2 (<1%)
Melanoma	2 (<1%)
Adrenal glands	1 (<1%)
Endometrium	1 (<1%)
Ewing sarcoma	1 (<1%)
Medulloblastoma	1 (<1%)
Nasopharynx	1 (<1%)
Oropharynx	1 (<1%)
Pancreas	1 (<1%)
Parotid	1 (<1%)
Testicles	1 (<1%)
Thymus	1 (<1%)
Haematologic malignancies	47 (17.28%)
Hodgkin lymphoma	22 (8.1%)
Non-Hodgkin lymphoma	10 (3.7%)
Multiple myeloma	6 (2.2%)
Acute myeloid leukaemia	3 (1.1%)
Myelodysplastic syndrome	3 (1.1%)
Chronic myeloid leukaemia	2 (<1%)
Hairy cell leukaemia	1 (<1%)
Unknown primary	2 (<1%)

*n*, frequency; %, percentage

**Table 3.** Challenges faced by cancer patients.

	Faced the challenge *n* (%)	Did not face the challenge *n* (%)
Availability of non-chemotherapy medications (i.e., Aspirin, Paracetamol, Hypertension and Diabetes medications...)	194 (71.3%)	78 (28.7%)
Availability of chemotherapy medications	135 (49.6%)	137 (50.4%)
Increase of the consultation fees with your Doctor	117 (43%)	155 (57%)
Transportation problems from residence to hospital	106 (39%)	166 (61%)
Deferral of radiotherapy/chemotherapy dates	86 (31.6%)	186 (68.4%)
Difficulty in maintaining precautionary measures (social distancing, wearing facemask etc...)	69 (25.4%)	203 (74.6%)
Are you able to reach your doctor online (i.e., WhatsApp, Phone calls etc…)	46 (15.8%)	226 (83.2%)
Long waiting hours beyond appointment time	35 (12.9%)	237 (87.1%)
Availability of hospital beds	34 (12.5%)	238 (87.5%)

*n*, frequency; %, percentage

**Table 4.** Challenges in private compared to public hospitals. Statistically significant values with *p* < 0.05 are highlighted in bold.

	Location of hospitalisation		*p*-value
	Private hospitals *n* (%)	Public hospital *n* (%)	
Transportation problems from residence to hospital	73 (33.5%)	33 (61.1%)	**<0.001**
Are you able to reach your doctor online (i.e. WhatsApp, Phone calls etc…)	30 (13.8%)	16 (29.6%)	**<0.001**
Difficulty in maintaining precautionary measures (social distancing, wearing facemask etc…)	54 (24.8%)	15 (27.8%)	0.650
Deferral of radiotherapy/chemotherapy dates	60 (27.5%)	26 (48.2%)	**0.004**
Availability of chemotherapy medications	96 (44.0%)	39 (72.2%)	**<0.001**
Availability of non-chemotherapy medications (i.e. Aspirin, Paracetamol, Hypertension and Diabetes medications...)	156 (71.6%)	38 (70.4%)	0.863
Long waiting hours beyond appointment time	29 (13.3%)	6 (11.1%)	0.667
Availability of hospital beds.	27 (12.4%)	7 (13.0%)	0.909
Increase of the consultation fees with your Doctor.	92 (33.8%)	25 (9.2%)	0.587

*n*, frequency; %, percentage of challenge when present

**Table 5.** Patients’ responses to Knowledge Scale.

Knowledge items	Correct	Wrong	Don’t know
1- Infected children showing no symptoms can spread the virus to others (Agree)	161 (59.2)	41 (15.1)	70 (25.7)
2- COVID-19 is always accompanied by a loss of taste and smell, in 100% of people who contract the virus (Disagree)	111 (40.8)	117 (43.0)	44 (16.2)
3- COVID-19 can spread through cough (Agree)	236 (86.8)	15 (5.5)	21 (7.7)
4- COVID-19 can spread through food, through the digestive system (Disagree)	101 (37.1)	90 (33.1)	81 (29.8)
5- Hot beverages protect from contracting COVID-19 (Disagree)	90 (33.1)	121 (44.5)	61 (22.4)
6- Eating garlic protects from contracting COVID-19 (Disagree)	73 (26.9)	132 (48.5)	67 (24.6)
7- COVID-19 can spread through the water of swimming pools (Disagree)	67 (24.6)	111 (40.8)	94 (34.6)
8- Wearing a face shield without a mask protects from contracting COVID-19 (Disagree)	169 (62.1)	56 (20.6)	47 (17.3)

Agree/Disagree in the parentheses denotes the intended correct answer

**Table 6.** Factors associated with better knowledge towards COVID-19.

	Knowledge			
	Below average *n* (%)	Above average *n* (%)	*p*-value	Adjusted OR (95%CI)
Age categories				
≤57 years	65 (43.0%)	72 (59.5%)	0.079	0.61 (0.34–1.06)
>57 years	86 (57.0%)	49 (40.5%)		
Intent of treatment				
Curative	139 (92.1%)	119 (98.3%)	0.144	0.31 (0.05–1.25)
Palliative	12 (7.9%)	2 (1.7%)		
Were you diagnosed with COVID-19?				
Yes	30 (19.9%)	38 (31.4%)	0.207	0.67 (0.35–1.25)
No	120 (79.5%)	83 (68.6%)		
Education status				
Illiterate	30 (19.9%)	6 (5.0%)	Reference group	
Elementary school	87 (57.6%)	45 (37.2%)	0.079	2.45 (0.95–7.22)
High school graduate	27 (17.9%)	41 (33.9%)	**<0.001**	6.32 (2.31–19.67)
College graduate	7 (4.6%)	29 (24.0%)	**<0.001**	19.27 (5.64–77.31)
Location of hospitalisation				
Public hospital	31 (20.5%)	23 (19.0%)	0.755*	
Private hospitals	120 (79.5%)	98 (81.0%)		
Are you worried about contracting COVID-19				
Minimally worried	55 (36.4%)	29 (24.0%)	Reference group	
Average level of worry	40 (26.5%)	46 (38.0%)	0.129	1.74 (0.85–3.59)
Very much	56 (37.1%)	46 (38.0%)	0.257	1.47 (0.76–2.9)
Which do you fear the most currently?				
COVID-19	42 (27.8%)	24 (19.8%)	0.280**	
Cancer	86 (57.0%)	82 (67.8%)		
Both equally	18 (11.9%)	13 (10.7%)		
None	5 (3.3%)	2 (1.7%)		
Can chemotherapy lower your immunity and increase the chance of health-related risks due to COVID-19?				
Yes	111 (73.5%)	95 (78.5%)	0.527	0.798 (0.40–1.61)
No	38 (25.2%)	22 (18.2%)		
Don’t know	2 (1.3%)	4 (3.3%)		

All variables listed above were included in the multivariate analysis.

*p*-value < 0.05 is considered to be significant

*n*, frequency; %, percentage

* Univariate analysis

** Chi-squared test

**Table 7.** Perspective about cancer and COVID-19.

	*n* (%)
Are you worried about contracting COVID-19	
Minimally worried	84 (30.9%)
Average level of worry	86 (31.6%)
Very much	102 (37.5%)
Can chemotherapy lower your immunity and increase the chance of health-related risks due to COVID-19?	
Yes	206 (75.7%)
No	60 (22.1%)
Don’t know	6 (2.2%)
Which do you fear the most currently?	
COVID-19	66 (24.3%)
Cancer	168 (61.8%)
Both	31 (11.4%)
None	7 (2.6%)
What preventive measures are you taking for COVID 19 at home?	
Facemask and social distancing only	36 (13.2%)
Self-quarantine only	65 (23.9%)
Both	76 (28%)
None of the above/other measures	95 (34.9%)

*n*, frequency; %, percentage

## Supplementary material

Initially, 15 statements were selected after reviewing the Centers for Disease Control and Prevention and the World Health Organization’s websites. A pilot study was then conducted on 30 patients from a single centre at Dar El Amal University Hospital in Baalbek, Lebanon. Reliability statistics were then conducted on the responses of these 30 participants, which yielded a Cronbach’s Alpha value of 0.683 (See Supplementary Table 1). As a Cronbach’s Alpha value of 0.7 and higher can be considered as reliable, statements numbered 4, 6, 7, 13 and 15 from Supplementary Table 1 were removed in order to increase Cronbach’s Alpha to 0.707 which is acceptable (See Supplementary Table 2).

Our study was conducted with the scale shown in Supplementary Table 2 including ten items in total.

Two items (2 and 10) were removed later on in the study as they were shown not to be accurate. Item number 2 was removed as runny nose can be a major symptom of COVID-19, and Item number 10 was removed as COVID-19 can present with hair loss due to stress like any other infection.

## References

[1] Liang W, Guan W, Chen R (2020). Cancer patients in SARS-CoV-2 infection: a nationwide analysis in China. Lancet Oncol.

[2] Garassino MC, Whisenant JG, Huang LC (2020). COVID-19 in patients with thoracic malignancies (TERAVOLT): first results of an international, registry-based, cohort study. Lancet Oncol.

[3] Al-Quteimat O, Amer A (2020). The impact of the COVID-19 pandemic on cancer patients. Am J Clin Oncol.

[4] Addeo A, Friedlaender A (2020). Cancer and COVID-19: unmasking their ties. Cancer Treat Rev.

[5] Fillmore NR, La J, Szalat RE (2020). Prevalence and outcome of COVID-19 infection in cancer patients: a national veterans affairs study. JNCI: J Natl Cancer Inst.

[6] Lee LYW, Cazier JB, Starkey T (2020). COVID-19 prevalence and mortality in patients with cancer and the effect of primary tumour subtype and patient demographics: a prospective cohort study. Lancet Oncol.

[7] Yang F, Shi S, Zhu J (2020). Clinical characteristics and outcomes of cancer patients with COVID‐19. J Med Virol.

[8] Torre L, Siegel R, Ward E (2015). Global cancer incidence and mortality rates and trends – an update. Cancer Epidemiol Biomarkers Prev.

[9] Kutikov A, Weinberg D, Edelman M (2020). A war on two fronts: cancer care in the time of COVID-19. Ann Intern Med.

[10] (2020). Surveillance in Lebanon. https://www.moph.gov.lb/en/Pages/2/194/surveillance-data#/en/Pages/2/24870/novel-coronavirus-2019-.

[11] (2021). World Bank. https://www.worldbank.org/en/country/lebanon/publication/lebanon-economic-monitor-spring-2021-lebanon-sinking-to-the-top-3.

[12] Das M (2021). Lebanon faces critical shortage of drugs. Lancet Oncol.

[13] Sturgis P, Roberts C, Smith P (2014). Middle alternatives revisited: how the neither/nor response acts as a way of saying ‘I don’t know’?. Sociol Methods Res.

[14] Fornell C, Larcker DF (1981). Evaluating structural equation models with unobservable variables and measurement error. J Mark Res.

[15] Carmines E, Zeller R (1979). Reliability and Validity Assessment.

[16] El Saghir NS, Khalil MK, Eid T (2007). Trends in epidemiology and management of breast cancer in developing Arab countries: a literature and registry analysis. Int J Surg.

[17] (2021). Lebanon | UNESCO UIS. http://uis.unesco.org/en/country/lb].

[18] Mitra M, Basu M (2020). A study on challenges to health care delivery faced by cancer patients in India during the COVID-19 pandemic. J Prim Care Community Health.

[19] Li H, Bailey A, Huynh D (2020). YouTube as a source of information on COVID-19: a pandemic of misinformation?. BMJ Glob Health.

[20] Ghosh J, Ganguly S, Mondal D (2020). Perspective of oncology patients during COVID-19 pandemic: a prospective observational study from India. JCO Glob Oncol.

